# Low arousal threshold: a common pathophysiological trait in patients with obstructive sleep apnea syndrome and asthma

**DOI:** 10.1007/s11325-022-02665-4

**Published:** 2022-07-30

**Authors:** Caterina Antonaglia, Giovanna Passuti, Fabiola Giudici, Francesco Salton, Barbara Ruaro, Dejan Radovanovic, Marco Confalonieri

**Affiliations:** 1 Pulmonology Department, University Hospital of Cattinara, Strada di Fiume 447, Trieste, Italy; 2 Pulmonology Department, Bellaria Hospital, Bologna, Italy; 3 Department of Medicine, Surgery and Health Science, University of Trieste, Trieste, Italy; 4 Pulmonary Unit, Luigi Sacco University Hospital, University of Milan, Milano, Italy

**Keywords:** Obstructive sleep apnea, aLow arousal threshold, Asthma and OSA, Alternative overlap syndrome

## Abstract

**Introduction:**

Obstructive sleep apnea syndrome (OSAS) and asthma are two diseases with a high epidemiological impact that may often coexist. Both diseases have underlying pathogenic mechanisms (chronic inflammation, genetic predisposition, etc.); it is still unclear whether or not their coexistence is due to a specific pathophysiological factor. In the literature, the pathogenesis of OSAS has four pathophysiological traits: one or more anatomical predisposing factors, a low arousal threshold (low AT), high loop gain, and poor muscle responsiveness. In this study, we hypothesized that a low AT is a common pathophysiological factor in OSAS and asthma.

**Methods:**

A retrospective study of patients attending the Pulmonology Unit of the University Hospital of Trieste was carried out. Low AT was predicted on the bases of the following polysomnography features, as previously shown by Edwards et al.: an AHI of < 30 events/h, a nadir SpO2 of > 82.5%, and a hypopnea fraction of total respiratory events of > 58.3%.

**Results:**

Thirty-five patients with asthma and OSAS and 36 with OSAS alone were included in the study. Low AT was present in 71% of patients affected by asthma and OSAS (25 patients out of 35) versus 31% (11 patients out of 36) of patients affected by OSAS alone with a statistically significant difference (*p* = 0.002) between the two groups. Stratifying for BMI and OSAS severity, the difference between groups remained statistically significant.

**Conclusions:**

This is the first study to describe specific polysomnographic characteristics of patients affected by asthma and OSAS. A low AT may well be the pathophysiological factor common to the two diseases. If confirmed by other studies, this finding could lead to the presence of asthma and OSAS in the same individual being considered a syndrome with a common pathophysiological factor.

## Introduction

### OSAS and asthma

Asthma and obstructive sleep apnea syndrome (OSAS) are two highly prevalent diseases that may coexist in some patients. Indeed, there is a 35% prevalence of asthma in adults with OSAS and patients with asthma run a higher risk of developing OSAS than the general population (39.5% vs 27.2%) [1]. OSAS is more prevalent in severe asthma than moderate or well-controlled asthma with a prevalence from 88 to 95% [2]. Epidemiological data suggest that the coexistence of OSAS and asthma has an adverse impact on health outcomes [3]. Asthma and OSAS overlap with similar comorbidities and underlying pathophysiology, potentiating the two conditions. The most common comorbidities associated with both diseases are gastro-esophageal reflux disease (RGE), rhino sinusitis, and obesity [4]. Neuromechanical reflex bronchoconstriction, local and systemic inflammation, the indirect effect on dyspnea of OSAS-induced cardiac dysfunction, angiogenesis, and leptin-related airway changes may all play a common mechanistic role in linking both disorders [5]. OSAS and asthma coexist not only due to these common comorbidities, but also because they could have a common pathophysiological mechanism [1, 6].

### OSAS pathogenesis and low arousal threshold

In recent years, it is clear that the anatomical predisposition factor is present in all patients as a predisposition factor (in 30% of patients as the only factor), but in 70% of cases, there is an associated non-anatomical factor, responsible for a different phenotype of the disease [7].

The four pathophysiological factors, involved in the pathogenesis of OSAS, that can contribute in a different way are anatomical factors (obesity, craniofacial conformations of reduced dimensions, laxity of the soft palate or macroglossia, etc.); instability of ventilatory control, also known as high loop gain; neuromuscular inefficiency of the dilator muscles of the upper airways also known as poor muscle responsiveness; increased propensity for nocturnal awakenings due to respiratory stimuli; or a reduced awakening threshold, also known as low arousal threshold (low AT).

The latter factor seems to be present in 30–50% of all patients with OSAS [8]. AT refers to the neuromuscular-mechanical pressure present at the end of an apnea–hypopnea event, responsible for awakening from sleep–arousal [9]. AT can be quantified only invasively by an epiglottic or esophageal pressure catheter [9]. A recent study by Edwards et al. reported that low AT could be estimated non-invasively through a following clinical score which attributes a point for each criterion met between: (AHI < 30 events/hour) + (nadir SpO2 > 82.5%) + (hypopnea fraction > 58.3%). A score of ≥ 2 predicts a low AT in OSAS patients, with high sensitivity and specificity (80.4% and 88% respectively) [10]. Also, other authors support the idea that it is possible to identify which of the non-anatomical factors contributes most to the pathogenesis of OSAS in that single patient, or in a particular subgroup, on the basis of the characteristics of the polysomnographic trace (or the cardiorespiratory monitoring trace) [11].

Indeed, it is believed that low AT contributes to the pathology of OSAS since the repeated awakenings determine destabilizing effects, such as (1) the absence of sufficient time for the respiratory drive to recruit the pharyngeal muscles and reopen the airways before arousal; (2) a reduced partial pressure of carbon dioxide, promoting dynamic ventilatory instability, which contributes to the perpetuation of the consequent respiratory events; and (3) a fragmentation of sleep, which does not allow the individual to achieve slow-wave sleep, i.e., to stabilize sleep [9, 12]. Therefore, it may be presumed an individual with low AT wakes up before a severe gas exchange abnormality (reduced SpO2) has developed. Similarly, those with better anatomy, i.e., a less severe airflow obstruction and a higher hypopnea fraction, should be more likely to have a non-anatomical underlying factor for their OSAS, such as a low AT [13]. However, it has not yet been clearly defined which factors are responsible for the AT reduction, and, to date, both chronic sleep fragmentation and intermittent hypoxia have been implicated [10]. Eckert et al. highlighted not only the inverse relationship between low AT, BMI, and AHI but also how the proposed clinical score predicts a prevalence of low AT in non-obese OSAS patients, where the anatomical factor seems to be less relevant [14].

The fact that symptoms worsen or are prevalent at night in asthmatic patients suggests the presence of a contributing factor during sleep which is connected to the presentation or aggravation of the disease. Indeed, an asthmatic patient often has poor quality sleep, especially if they suffer from severe or uncontrolled asthma, which is characterized by nocturnal awakenings and consequent sleep fragmentation [15]. This may affect the achievement of phase N3 and can contribute to ventilatory instability [16]. There may also be moments of rapid and shallow breathing, which determine a CO2 fluctuation and ventilatory instability, pathogenetic elements that recall the characteristics of OSAS patients with a low AT [17].

This led us to hypothesize that there may be a common pathogenetic element in patients with both asthma and OSAS, i.e., the presence of a low AT or that this factor is at least prevalent in this subgroup of patients compared to individuals with OSAS without asthma. This study evaluated cardiorespiratory monitoring findings, focusing on the low AT, in patients with OSA and asthma, and the prevalence of these features in patients with OSAS and asthma compared to patients with only OSAS.

## Methods

### Study design

This retrospective study was carried out at the Pulmonology Unit of the University Hospital of Trieste, Italy, in September 2020 as a follow-up done from July 2017 to September 2020. The diagnosis of OSAS was made by the use of a home sleep apnea test (HSAT) SOMNOlab 2 (Weinmann, Hamburg, Germany). Each patient had cardiorespiratory monitoring or HSAT up to July 2017 and pulmonary function testing within 5 years from the date of their nocturnal exam. Only patients with an apnea–hypopnea index (AHI) ≥ 5 events/h and symptoms or signs were enrolled (as the OSAS diagnostic criteria require) [18, 19].

Apnea and hypopnea were defined as respectively cessation or reduction of airflow lasting > 10 s. For hypopnea, our sleep center used the criteria of a > 50% reduction in airflow, accompanied by a > 3% decrease in SpO2. The study design was approved by the referral Ethics Committee. Given the retrospective nature of the study and the use of anonymous patient data, requirements for informed consent were waived.

The diagnosis of asthma was made according to the Gina Guidelines on the basis of the patient’s clinical history or current presence of respiratory signs and symptoms consistent with asthma, along with the objective demonstration of variable airflow obstruction confirmed by spirometry or peak expiratory flow monitoring [20]. Ont the day on which they performed HSAT, all patients with asthma included in our study had an asthma control test (ACT) between 20 and 25. Also, only 23 patients out of 35 performed spirometry at the time of HSAT. A total of 36 patients with a diagnosis of OSAS (group 1) and 35 patients with a diagnosis of both asthma and OSAS (group 2) were included. Patients with other chronic respiratory diseases, e.g., chronic obstructive pulmonary disease, interstitial lung diseases, or other respiratory diseases of any cause were excluded.

Assessments were made to determine if there were any statistically significant differences (*p* < 0.05) in the prevalence of low AT both between the two study groups and the subgroups stratified for BMI (< 30 kg/m^2^ or ≥ 30 kg/m^2^) and OSAS severity (mild OSAS if 5 ≤ AHI < 15, moderate OSAS if 15 ≤ AHI < 30, severe OSAS if AHI ≥ 30). Further analysis involved the association with low AT and Epworth Sleepiness Scale (ESS) and low AT and the FEV1 value measured before and after OSAS diagnosis and therapy, in association with gender and/or age (< 62 or ≥ 62 years old).

The clinical scoring system described by Edwards et al [12]. was used to predict the presence of low arousal threshold. One point was assigned to each of the following three criteria: an AHI of < 30 events/h, a nadir SpO2 of > 82.5%, and a hypopnea fraction of total respiratory events of > 58.3%. A total score of ≥ 2 was defined as a low arousal threshold.

### Statistical analysis

The Shapiro–Wilk test was applied to quantitative (continuous) variables to assess distribution normality. Continuous variables were reported as median with range (minimum–maximum) or mean and standard deviation (SD). Qualitative (categorical) variables were reported as absolute frequencies and/or percentages. Continuous variables were compared between the two groups (OSAS vs OSAS + asthma) by Student’s t-test or the Mann–Whitney test, depending on the data distribution. Categorical variables were compared by the chi-square test or Fischer’s exact test, as appropriate. The Spearman correlation coefficient was calculated to assess any linear correlation between BMI and AHI, BMI and ESS, and between AHI and ESS. All statistical analyses were carried out by an R system for statistical computing (Ver. 4.0.2; R Development Core Team, 2020). All tests were two-tailed and statistical significance was set at a *p*-value of < 0.05.

## Results

**Fig. 1:**
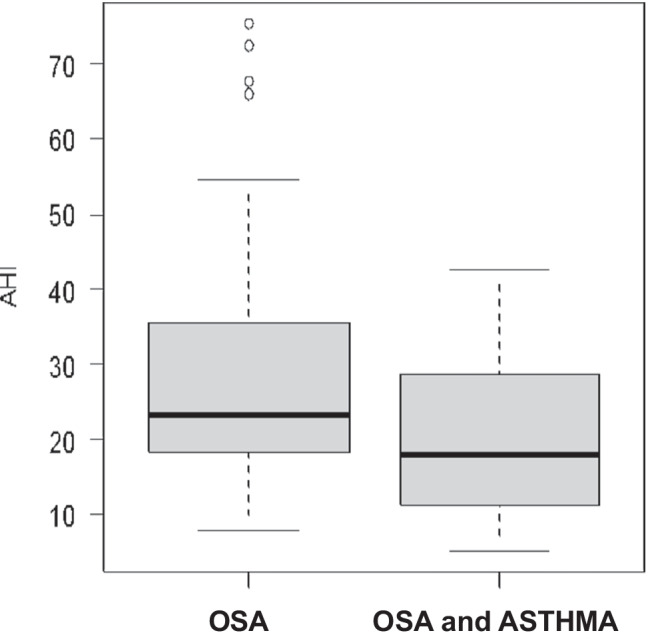
AHI distribution

**Fig. 2:**
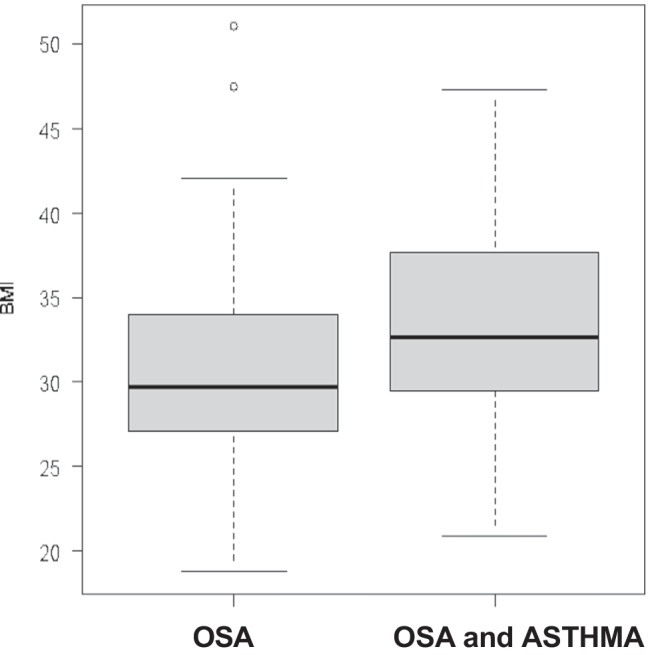
BMI distribution

**Fig. 3:**
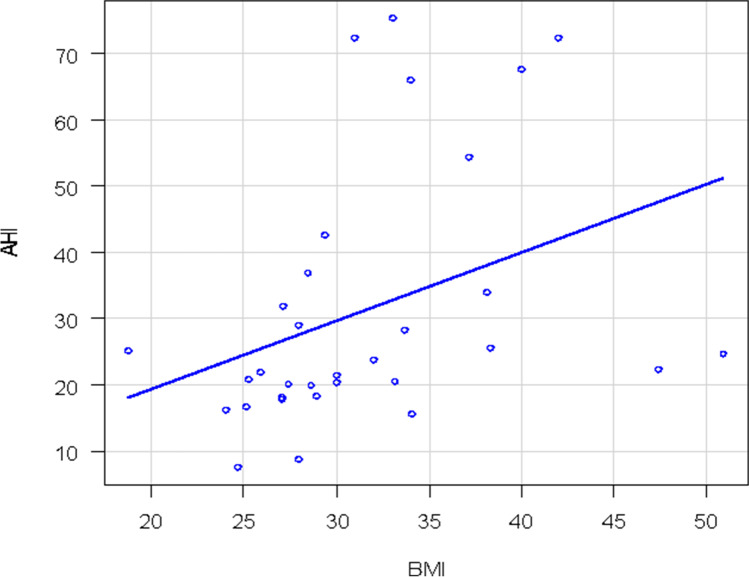
Correlation between AHI and BMI in group 1

**Fig. 4:**
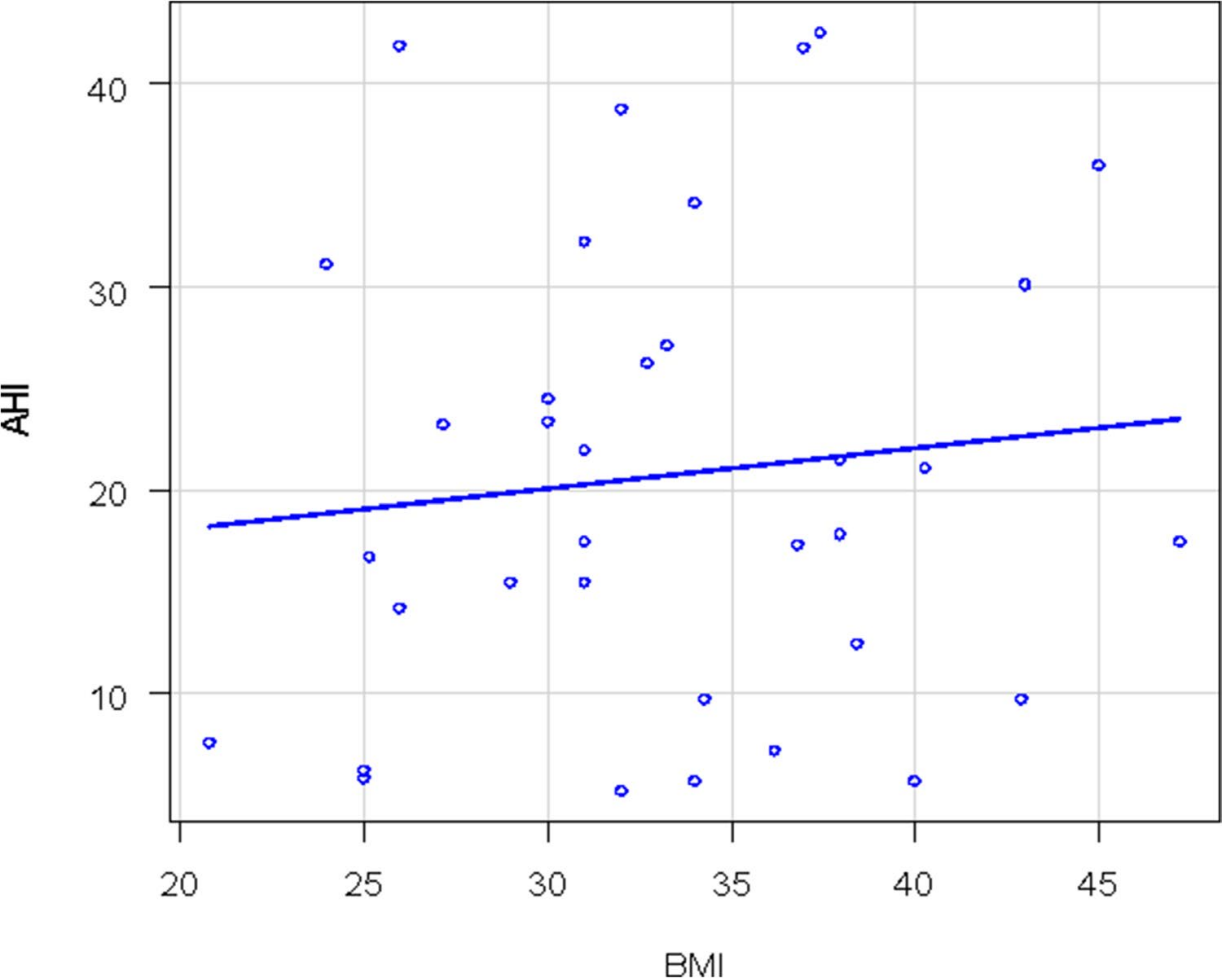
Correlation between AHI and BMI in group 2

**Fig. 5:**
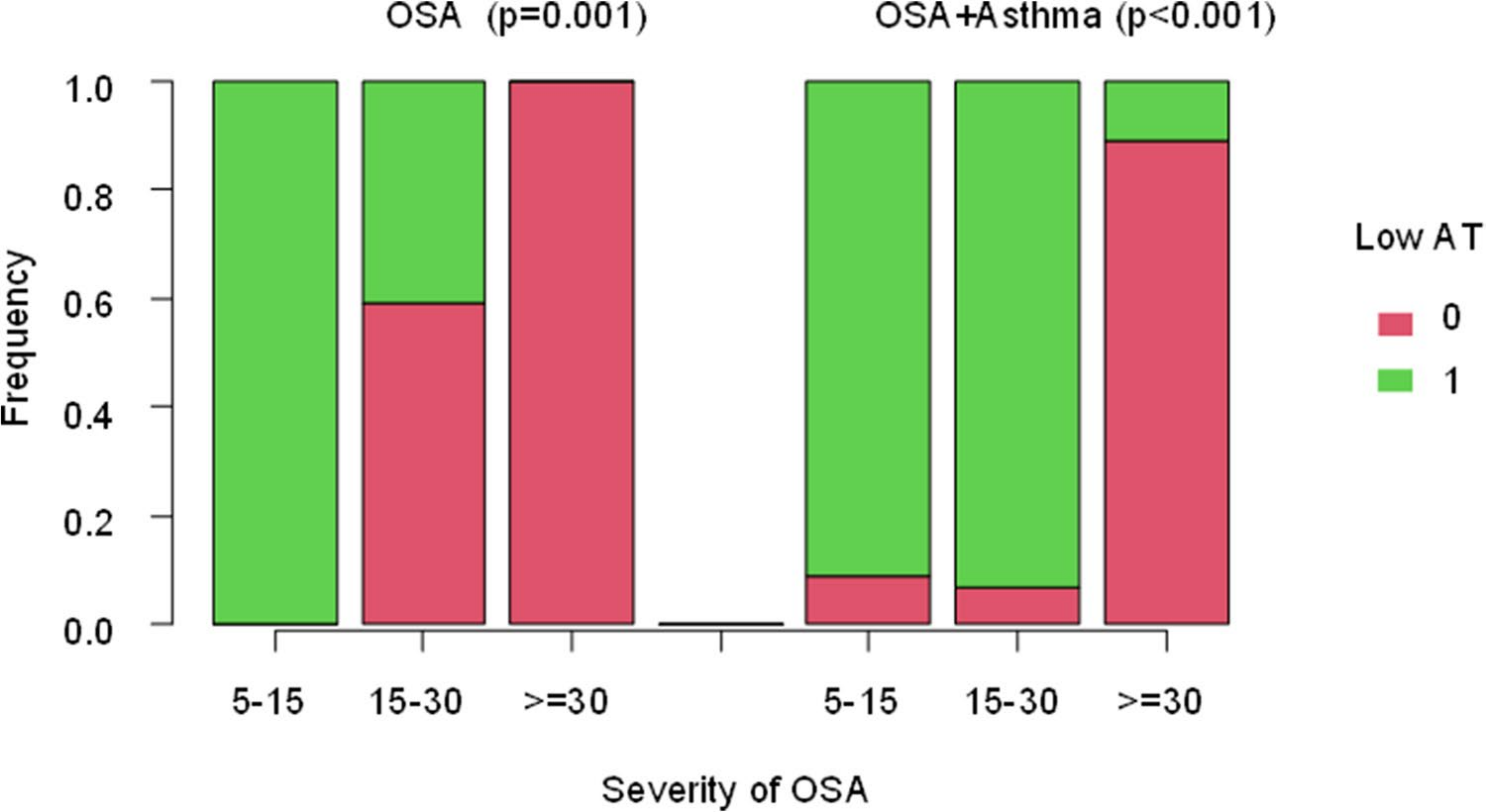
Low AT and severity of OSA

Of 71 patients enrolled according to the previous mentioned criteria, 36 had OSAS only (group 1) and 35 had both OSAS and asthma (group 2). The main characteristics of the patients are illustrated in Table 1. There was a statistically significant difference in the mean AHI distribution between the two groups (*p* = 0.02). The median AHI value in group 1 was 23 and 17.8 for group 2. Group 1 patients had a more severe OSAS than group 2 (Fig. 1). This difference was present only for obese patients (*p* = 0.04) and not for non-obese patients (*p* = 0.13). Two patients in group 1 (6%) had mild OSAS, 22 (61%) had moderate OSAS and 12 had (33%) severe OSAS. Only 23 patients of 35 with asthma performed a spirometry at the time of HSAT with a mean value of FEV1 of 85%. Eleven patients in group 2 (31%) had mild OSAS, 15 (43%) had moderate OSAS, and 9 (26%) had severe OSAS. There were 16/36 (44%) non-obese patients (BMI < 30 kg/m^2^) vs 16/36 (44%) obese patients (BMI ≥ 30 kg/m^2^) in group 1, the BMI of 4 patients was unknown. A total of 9/35 patients (26%) in group 2 were non-obese and 26/35 (74%) were obese while the BMI was unknown in 3 patients (Fig. 2). There was a significant correlation between AHI and BMI only in group 1: as the BMI increases, so do the AHI values (Fig. 3). In group 2, there was no correlation between AHI and BMI in obese and also in non-obese patients. Assessment by the Edwards et al. clinical score [10] revealed a total of 25/35 patients (71%) in group 2 had low AT and 11/36 (31%) had low AT in group 1. There was a statistically significant prevalence of low AT in group 2 (*p*-value = 0.001), but this result was statistically significant only in the obese patients. A statistically significant difference was observed in the prevalence of low AT between the two groups in patients with moderate OSAS (15 ≤ AHI < 30), with a *p*-value = 0.01, but not in patients with mild OSAS (*p*-value = 1) or severe OSAS (*p*-value = 0.43). Data are shown in Figs. 4 and 5

**Table 1:** The main patient characteristics for group 1 and group 2

Variable	OSA (group 1) (*n* = 36)	OSA + asthma (group 2) (*n* = 35)	*p*-value
Age (years)			
Mean (SD)	61 (12)	63 (10)	*p*-value = 0.40
Gender (*N* %)			
Female	6 (17%)	13 (37%)	*p*-value = 0.05
Male	30 (83%)	22 (63%)	
BMI (kg/m^2^)			
Median (min–max)	29.7 (18.8–51.0)	32.7 (20.8–47.3)	*p* = 0.18
BMI (*N*, %)			
< 30	16 (50%)	9 (26%)	*p*-value = 0.04
≥ 30	16 (50%)	26 (74%)	
Epworth Sleepiness Scale (ESS)			
Median (min–max)	5 (1–17)	6.5 (1–11)	*p*-value = 0.48
Missing (*N*, %)	2 (8%)	17 (49%)	
Low AT			
0	25 (69%)	10 (29%)	*p*-value = 0.001
1	11 (31%)	25 (71%)	
AHI			
Median (min–max)	23.0 (7.7–75.3)	17.8 (5.2–42.6)	*p*-value = 0.02
AHI (*N*, %)			
5–15	2 (6%)	11 (31%)	*p*-value = 0.019
15–30	22 (61%)	15 (43%)	
≥ 30	12 (33%)	9 (26%)	
Nadir SatO2 (%)			
Median (nin–max)	79.5 (53–88)	84.0 (59–90)	*p*-value = 0.006
Nadir SatO2 > 82.5% (*N*, %)			
Yes	12 (33%)	26 (74%)	*p*-value = 0.001
Hypopnea fraction of total respiratory events (%)			
Median (min–max)	0.25 (0.003–0.94)	0.29 (0.000–0.95)	*p*-value = 0.19
F hypopneas > 58.3% (*N*, %)			
Yes	5 (14%)	7 (20%)	*p*-value = 0.49

There was a statistically significant association between low AT and BMI in group 1 but not in group 2 as shown in Table 2. There were lower ESS values in patients with low AT than in non-low AT patients in group 1, without statistical significance (medians 4 vs 8, respectively, *p*-value = 0.12). This trend was not evident in group 2 (*p*-value = 0.78). Data are shown in Table 3.

**Table 2:** Association between low AT and BMI

	Variables	BMI < 30	BMI ≥ 30	*p*-value
Group 1	Low AT = 0	8 (50%)	14 (88%)	*p*-value = 0.05
	Low AT = 1	8 (50%)	2 (13%)	
	Total	16	16	
Group 2	Low AT = 0	2 (22%)	8 (31%)	*p*-value = 1.00
	Low AT = 1	7 (78%)	18 (69%)	
	Total	9	16

**Table 3:** Association between low AT and AHI: in the two groups there is an association between low AT and AHI

	Variables	5 ≤ AHI < 15	15 ≤ AHI < 30	AHI ≥ 30	*p*-value
Group 1	Low AT = 0	0 (0%)	(59%)	12 (100%)	*p*-value = 0.001
	Low AT = 1	2 (100%)	(41%)	0 (0%)	
	Total	2	22	12	
Group 2	Low AT = 0	1 (9%)	1 (7%)	8 (89%)	*p*-value < 0.001
	Low AT = 1	10 (91%)	14 (93%)	1 (11%)	
	Total	11	15	9	

There was no statistically significant difference in the association between low AT and FEV1 measured at or near the diagnosis of OSAS and, therefore, before starting any therapy for OSAS (*p*-value = 0.7), nor between low AT and FEV1 assessed after starting OSAS therapy (*p*-value = 0.55).

## Discussion

The data obtained in this study suggested that patients with both OSAS and asthma (group 2) often had polysomnographic features of low arousal threshold, characterized by flow limitation, prevalent hypopnea, oxygen desaturation, and a less severe syndrome. There was a statistically more frequent observation of low arousal threshold in group 2 than in group 1.

The pathophysiological hypothesis is that there are several factors that lead to a faster and more superficial breathing with greater instability of the respiratory drive in asthmatic patients, and therefore, they have a predisposition to a low AT. Such mechanisms include nocturnal awakenings which disrupt sleep, increase latency to sleep or reduce total sleep time (especially in severe asthma), decrease functional residual capacity (FRC), especially during REM sleep and increase airway resistance at night (bronchoconstriction accentuated by the increase in cholinergic tone, especially in allergic asthma) [21]. The nocturnal breathing of asthmatic patients has characteristics related to hormonal, cytokine, vagal changes, etc., which induce sleep itself (a leukotriene peak at 4 am). Asthmatic patients have an increase in airway resistance, due to an increased nocturnal bronchoconstriction, which would seem to be associated with an increased activity of the respiratory muscles, recorded on electromyography (EMG), aimed at maintaining tidal volume stable at the expense of a reduction in inspiratory time and a small increase in respiratory rate. So, the secondary increase in central respiratory drive could contribute to the achievement of arousal. In patients with poorly controlled asthma nocturnal awakenings, sleep fragmentation and reduced total sleep time could contribute to a low AT, but also the presence of ventilatory instability [22]. All these features led us to hypothesize that low AT is a pathophysiological factor in patients with asthma and OSAS. Our results support the higher prevalence of low AT in OSA and asthma patients compared to controls.

Interestingly, our data confirmed this prevalence in obese asthmatic patients, differently from previous literature data that reported low AT was more frequently observed in non-obese patients [14]. This is noteworthy, in as much as it supports the hypothesis that it is asthma that contributes to the genesis of low AT, regardless of the absence of a state of obesity. Analyzing the distribution of AHI between two groups, it was observed that patients without asthma generally more marked in the obese patients (*p*-value = 0.03), i.e., non-asthmatic and obese patients are more frequently affected by severe OSAS. A brief awakening was more common following a relatively mild airway obstruction during sleep in patients with a low AT, leading to persistent respiratory instability [9] and, above all, they rarely reached a severe AHI.

However, our results for the non-asthmatic patients were in line with what is reported in the literature, i.e., obesity is linked to a greater OSAS severity. Indeed, visceral obesity is correlated with a greater deposition of peri-pharyngeal fat, which increases the mass load at the level of the upper airways and reduces the longitudinal traction strength of the trachea, leading to an increase in the collapse of the upper airways [23]. Therefore, obese patients with OSAS tend to have more severe OSAS than non-obese patients [24, 25].

As described in the literature, low AT is present in 30–50% of OSAS patients, especially in non-obese patients (BMI < 30 kg/m^2^) and those with mild/moderate OSAS (5 ≤ AHI < 30) [12].

We also observed a high prevalence of low AT in patients with mild OSAS and a low, almost absent, prevalence of low AT in patients with severe OSAS, in both groups. However, noteworthy was the fact that there was a statistically significant difference (*p*-value = 0.04) between the two groups as to the prevalence of low AT in patients with moderate OSAS (Fig. 5). There was a prevalence of low AT in asthmatic patients with moderate OSAS. The higher prevalence of low AT observed in asthmatic patients was not related to a selection bias of patients with less severe OSAS, as demonstrated by the fact there was no difference in the prevalence between two groups for patients with mild and severe OSAS. Therefore, our findings support the idea that patients with co-presence of OSA and asthma have a more frequent “phenotype with low AT” which contributes to the pathogenesis of OSAS then controls. As aforementioned, sleep in asthmatic patients is characterized by fluctuations in leukotriene levels, cholinergic activity, inflammation, and bronchoconstriction, leading to an increase in airway resistance, which some studies have reported to be associated with an increase in the EMG activity of the respiratory muscles [22], while others have attributed it to a small increase in respiratory rate and the tidal volume/inspiratory time ratio, suggesting an increased activity of the respiratory drive [26]. This element, associated with a fragmented sleep by arousal or with a reduction in the N3 sleep stage, would support the hypothesis that a low AT in asthmatic patients may play a predisposing role for the onset of OSAS. However, the literature on the association between low AT and increased daytime sleepiness is scanty. Indeed, it is known that the presence of low AT is most likely associated with a reduction in rest during the night and the need for sedatives, a finding that has been reported for the non-obese patient with OSAS, who frequently has a low AT [14]. However, to the best of our knowledge, no correlation between a low AT and increased daytime sleepiness has yet been confirmed.

This study used self-administered questionnaires to evaluate the severity of daytime sleepiness, using the Epworth Sleep Scale (ESS) and compared it in patients with and without a low AT in both groups. No statistically significant differences were observed even when the evaluation was made in association with BMI or OSAS severity (AHI). Our data showed that having a low AT did not appear to affect daytime sleepiness. However, the small sample size (53 patients) and the strong subjectivity of the ESS could have influenced these results.

Furthermore, there was no statistically significant difference between patients with or without low AT in group 2 as to the distribution of FEV1, measured before and after the use of CPAP. Different types of treatment for OSAS were not discriminated. Therefore, it would be interesting to distinguish the various types of treatment administered for OSAS and to carry out a study aimed at evaluating the effects of this therapy on lung function (in terms of FEV1). Indeed, currently, there is no consensus as to the specific effect various treatments may have on OSAS and asthma, although effectively treating and controlling OSAS seems to improve its clinical outcome as well as the outcome of associated bronchial asthma [27]. As reported in the literature, the presence of a low AT may have important implications for OSAS therapy. In particular, interventions concerning non-anatomical factors (such as non-muscle relaxant sedatives) aimed at increasing AT, to be used alone or in combination with other therapies (CPAP, mandibular advancement devices), could contribute to therapeutic success in some subgroups of non-obese patients with low AT, that seem to have poor CPAP adherence. [28]

Any confirmation of low AT as a pathophysiological factor underlying the coexistence of asthma and OSA may well lead to important implications in the therapeutic approach of these patients.

We are of the opinion that it would be both useful and appropriate to perform prospective studies to ascertain whether the optimization of asthma therapy is able to reduce arousals and increase AT in patients with OSAS and, consequently, also improve the severity of the OSAS. Indeed, to date, the factors contributing to low AT in OSAS patients have not yet been fully clarified, nor is the true association between low AT and bronchial asthma known, leaving the complex mechanisms underlying bronchial asthma and OSAS still to be clarified. The results of this study show that asthma is a comorbidity that contributes to low AT in patients with OSAS, meaning that this datum could be of help in future investigations into the pathogenesis of OSA in asthmatic patients.

We are aware that this study does have some limits. The main one is the small sample size and it would be interesting to verify if the statistically significant differences observed for the objectives studied hold fast for larger samples. Small sample size permits only univariate analyses, not accounting for potential confounders as for example gender and age. Another limitation is the use of the clinical score proposed by Edwards et al., as it has not yet been validated on a large scale [12]. However, the investigators also added that their clinical score requires validation in a large independent dataset and that prospective validation studies could also evaluate the efficacy of the current scoring criteria. [28]

Our measure of arousal threshold is based on HSAT data and not polysomnographic date with EEG. It was not possible to measure AT with invasive techniques during the standard polysomnographic study in order to verify the actual presence of low AT in patients with a clinical score of ≥ 2 according to the criteria proposed by Edwards et al. [10]. However, the re-analysis of the cardiorespiratory monitoring pattern performed for each patient with low AT confirmed the presence of the features, described by Bosi et al. [11], associated with the presence of a non-anatomical predisposing factor for OSAS.

Other studies would be needed, in particular AT in asthmatic patients without OSAS. It could also be useful, to clarify the role of AT and to try looking into the effect of CPAP therapy in our two groups of patients.

Two other important comorbidities between OSAS and asthma were not taken into consideration, i.e., gastroesophageal reflux disease and rhinitis. Therefore, whether or not the statistically significant difference was also confirmed in association with these two comorbidities was not evaluated. Another limit of our study is the definition of asthma control of our patients, even if all patients underwent nocturnal cardiorespiratory monitoring in the absence of exacerbated asthma. We defined control of Asthma by ACT.

Our study is in line with recent literature that support the idea that OSAS is a heterogeneous disorder in terms of its pathogenesis and clinical expression with different phenotypes. Asthma may influence the phenotype of OSAS by reducing arousal threshold such that the coexistence of asthma and OSAS could be considered a syndrome or a clinical phenotypic trait of OSAS.

## Data Availability

Data will be available on reasonable request to the corresponding author.
